# Supplementary material for: The molecular organization of differentially curved caveolae indicates bendable structural units at the plasma membrane
Source: Nat Commun. 2022 Nov 24;13:7234. doi: 10.1038/s41467-022-34958-3 (PMC9700719; doi:10.1038/s41467-022-34958-3)
Supplement: Supplementary file 1 — Supplementary Info [file 41467_2022_34958_MOESM1_ESM.pdf]

Supplementary Information

The molecular organization of differentially curved caveolae indicates bendable structural units at the plasma membrane.

Claudia Matthaeus[1], Kem A. Sochacki[1], Andrea Dickey[1], Dmytro Puchkov[2], Volker Haucke[2,3], Martin Lehmann[2], Justin W. Taraska[1*]

[1] Biochemistry and Biophysics Center, National Heart, Lung, and Blood Institute, National Institutes of Health, Bethesda, MD, USA *justin.taraska@nih.gov
[2] Leibniz-Forschungsinstitut für Molekulare Pharmakologie (FMP), Berlin, Germany
[3] Faculty of Biology, Chemistry and Pharmacy, Freie Universität Berlin, Berlin, Germany
*Correspondence should be addressed to Justin W. Taraska (justin.taraska@nih.gov)

Suppl. Figure 1: Representative PREM images of caveolae rosettes in various cell types.

Suppl. Figure 2: Analysis of the caveolar coat stripes in PREM images.

Suppl. Figure 3: Caveolae can be classified in low, medium and highly curved invagination types.

Suppl. Figure 4: Exact caveolae size measurement by STED microscopy.

Suppl. Figure 5: STED-CLEM overview of plasma membrane sheets showing clathrin and cavin1.

Suppl. Figure 6: Immature caveolae sites can be detected by STED-CLEM.

Suppl. Figure 7: Cavin localization at low curved caveolae after mild osmotic shock in HUVEC.

Suppl. Figure 8: Representative overview STED-CLEM images of endogenously stained cavin2 or 3 in HUVEC plasma membrane sheets treated with strong osmotic shock

Suppl. Figure 9: Cavin1, 2 and 3 localization to Caveolin1 in intact HUVEC after osmotic shock.

Suppl. Figure 10: Endogenous staining of Pacsin2 and EHBP1 at caveolae.

Suppl. Figure 11: Western Blot validation of pacsin2 and EHBP1 knockdown.

Suppl. Figure 12: Immunostaining of endogenous dynamin in MEFs did not show localization to caveolae.

Suppl. Figure 13: STORM-CLEM in HeLa and SK-MEL-2 cells did not show dynamin localization to caveolae.

Suppl. Figure 14: Western Blot validation of dynamin triple knockout

Suppl. Video1: Electron tomogram of MEF plasma membrane sheet including caveolae.

Suppl. Video2: Electron tomogram of low curved caveolae.

Suppl. Video3: Electron tomogram of medium curved caveolae.

Suppl. Video4: Electron tomogram of highly curved caveolae.

Source data are provided in Source Data file.

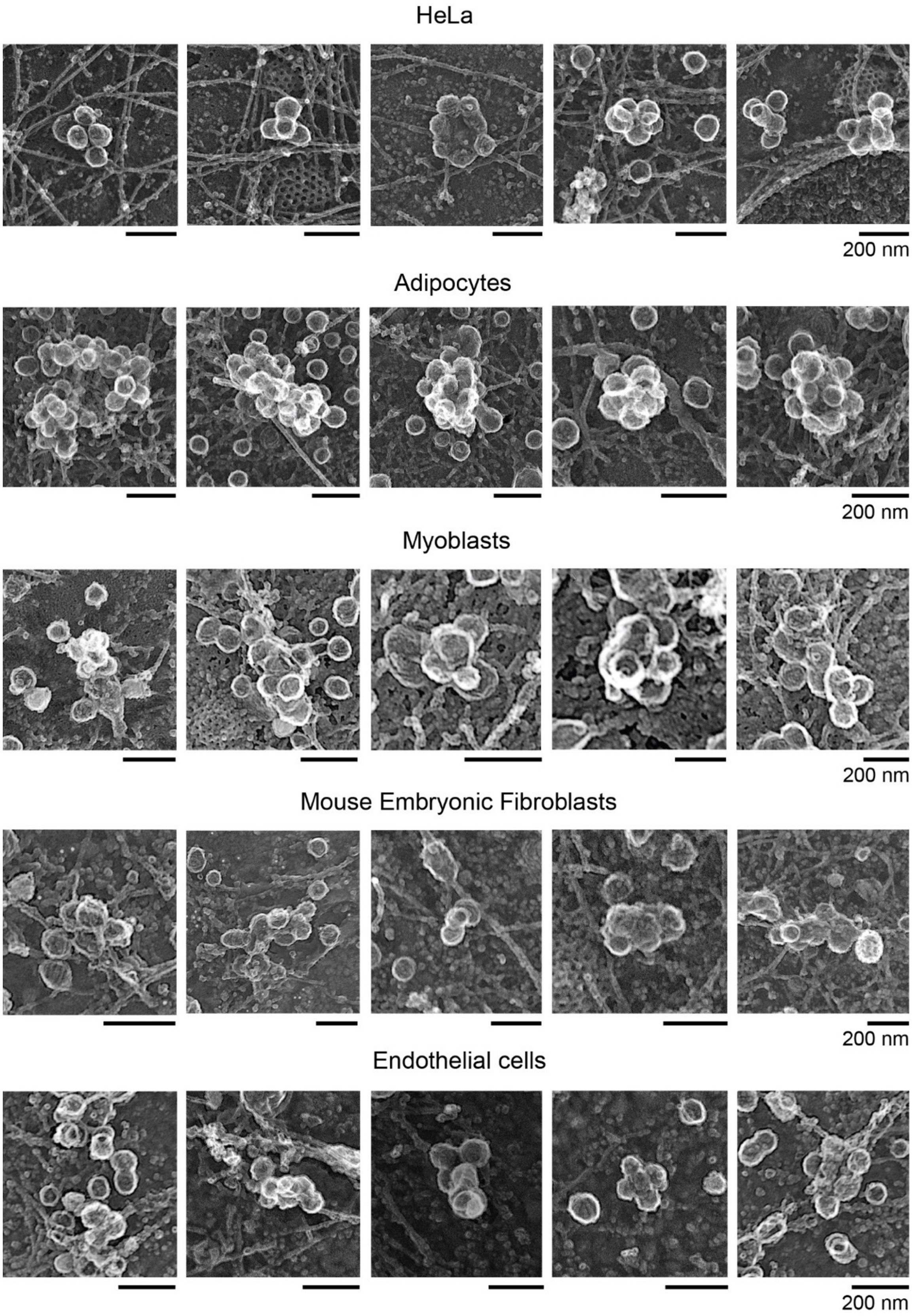
HeLa
200 nm
Adipocytes
200 nm
Myoblasts
200 nm
Mouse Embryonic Fibroblasts
200 nm
Endothelial cells
200 nm

Suppl. Figure 1: Representative PREM images of caveolae rosettes in various cell types. (3 independent experiments)

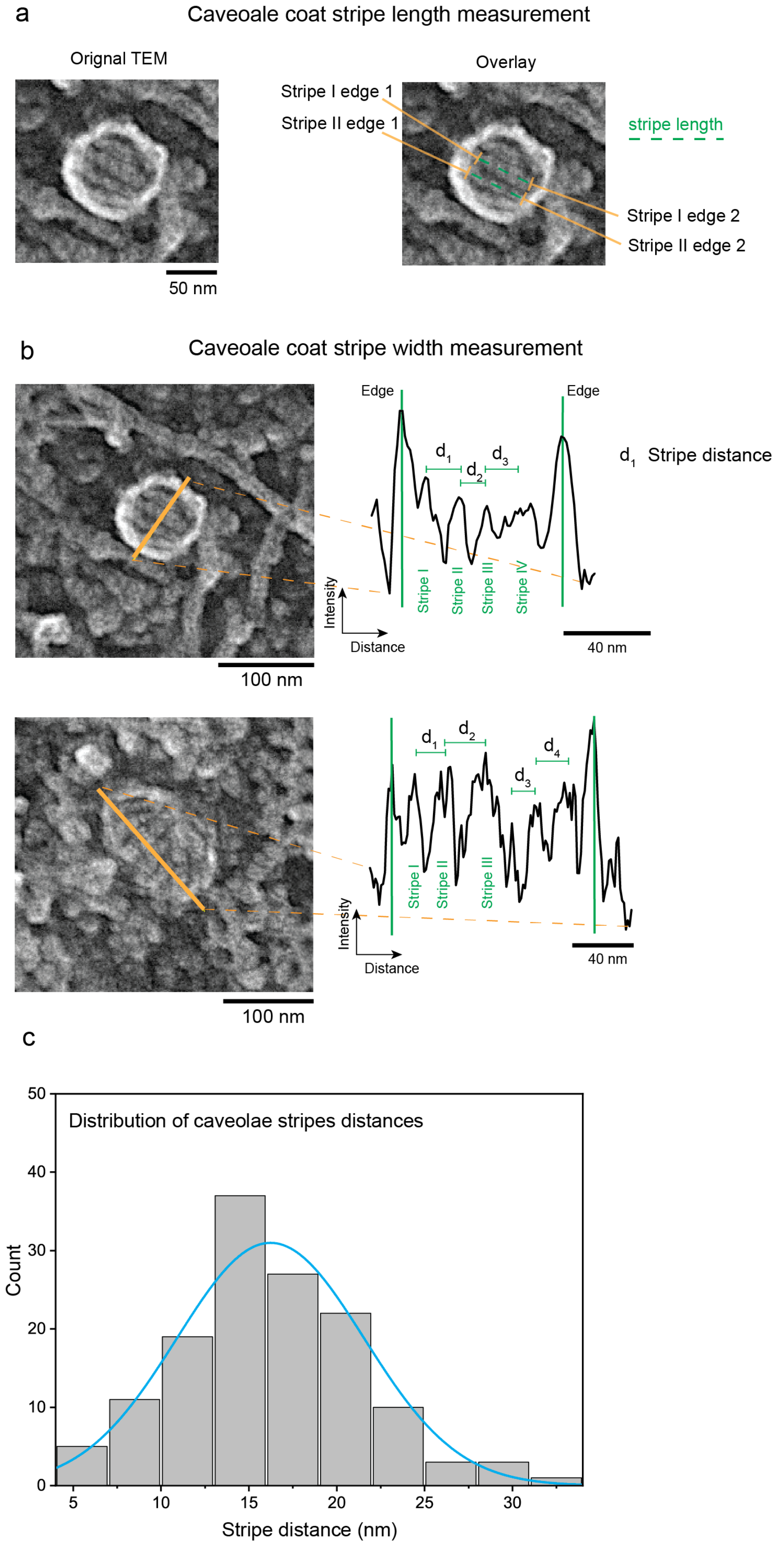

Suppl. Figure 2: Analysis of the caveolar coat stripes in PREM images.

(a) The length of the caveolar coat stripe was measured by ImageJ in which the distance (in nm) from one stripe edge to the other edge was measured (as indicated in the left overlay TEM image).

(b) Caveolae stripe width was estimated by measuring the distance between neighboring stripes in intensity plots of stripes within a caveolar domain. Maxima in the intensity plot illustrate stripes and minima the dark edge between neighboring stripes.

(c) Distribution of caveolae stripe distances measured in PREM of MEFs (n =138/5 cells, mean = 16.2 nm ± 0.5 nm; 3 independent experiments).

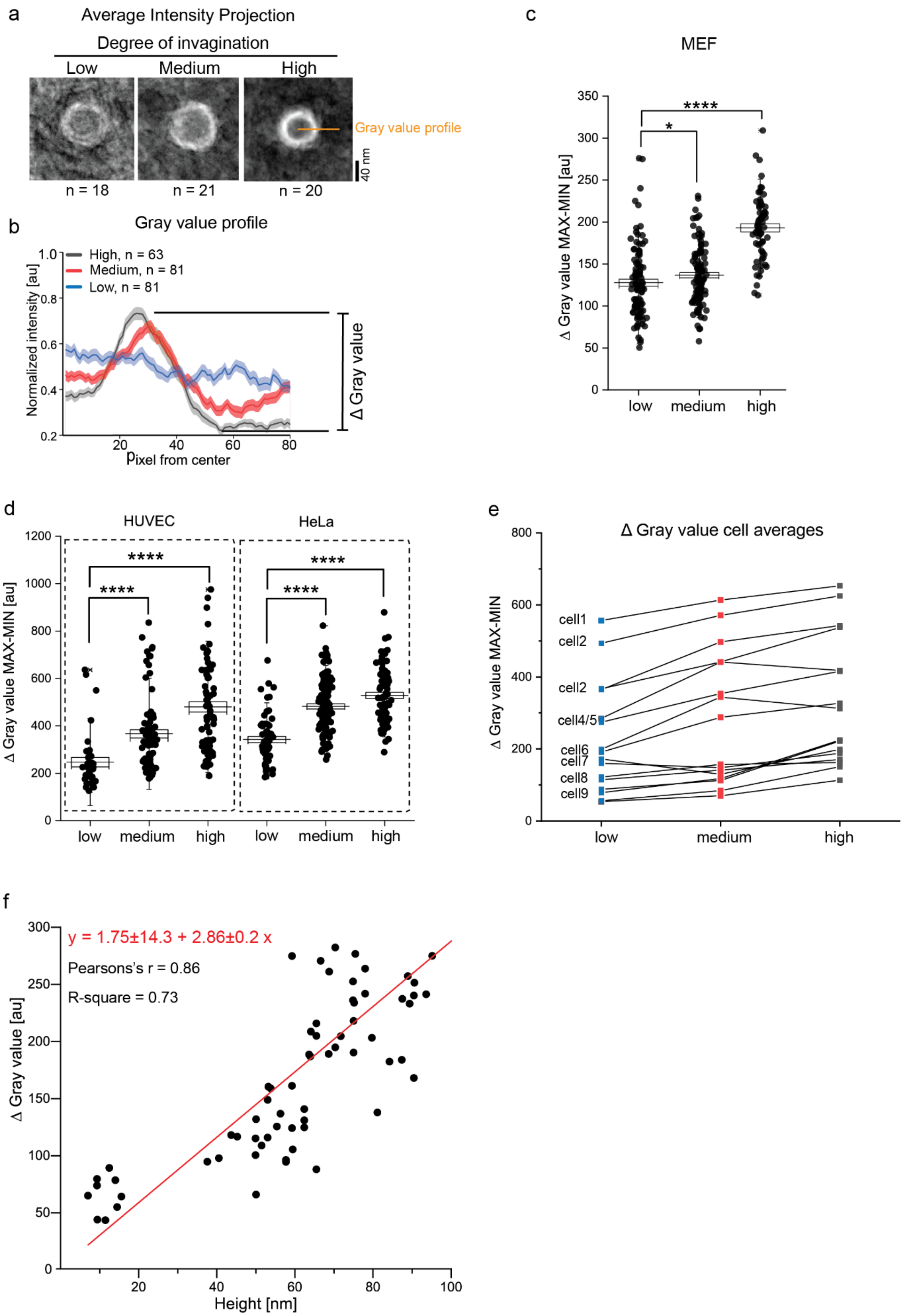

a
Average Intensity Projection
Degree of invagination
Low
Medium
High
Gray value profile
40 nm
n = 18
n = 21
n = 20
b
Gray value profile
High, n = 63
Medium, n = 81
Low, n = 81
Normalized intensity [au]
Δ Gray value
Pixel from center
c
MEF
Δ Gray value MAX-MIN [au]
low
medium
high
d
HUVEC
HeLa
Δ Gray value MAX-MIN [au]
low
medium
high
low
medium
high
e
Δ Gray value cell averages
Δ Gray value MAX-MIN
cell1
cell2
cell2
cell4/5
cell6
cell7
cell8
cell9
low
medium
high
f
y = 1.75±14.3 + 2.86±0.2 x
Pearsons's r = 0.86
R-square = 0.73
Δ Gray value [au]
Height [nm]

Suppl. Figure 3: Caveolae can be classified in low, medium and highly curved invagination types.

(a) Average intensity projection of 18 low, 21 medium, or 20 highly curved caveolae in MEFs.

(b) The intensity plot illustrates normalized electron intensity, mean ± SE from the center of the caveolae domain to its edge (gray value profile in a, caveolae number: n(low) = 63/4 cells, n(medium) = 81/4 cells, n(high) = 81/4 cells).

(c-d) Delta grey value between minimal and maximal grey value in intensity plot profile (as illustrated in b) of caveolae domains in MEFs (c), HUVEC and HeLa (d). Intensity plot profiles were un-biased measured from the center of caveolae to their edges (averaging of 12 radial scans/caveolae, caveolae number: MEF: n(low) = 103; n(medium) = 116, n(high) = 63, * P = 0.018, **** P = 3.1E-16, HUVEC: n(low) = 37; n(medium) = 80, n(high) = 71, **** P(medium) = 2.2E-7, P(high) = 3.5E-10, HeLa: n(low) = 63; n(medium) = 110, n(high) = 77, **** P(medium) = 1.18E-12, P(high) = 1.8E-15, 3 independent experiments, box plot indicates mean ± SE, whiskers show SD, each replicate is depicted, statistical difference was measured by two-sided Mann Whitney test).

(e) Average delta grey value per individual cell for low, medium or highly curved caveolae (n = 16 cells/9 independent experiments).

(f) Individual caveolae delta grey values (n = 75 caveolae/3 cells) were plotted against their corresponding heights measured in electron tomograms. Linear correlation trend is depicted in red.

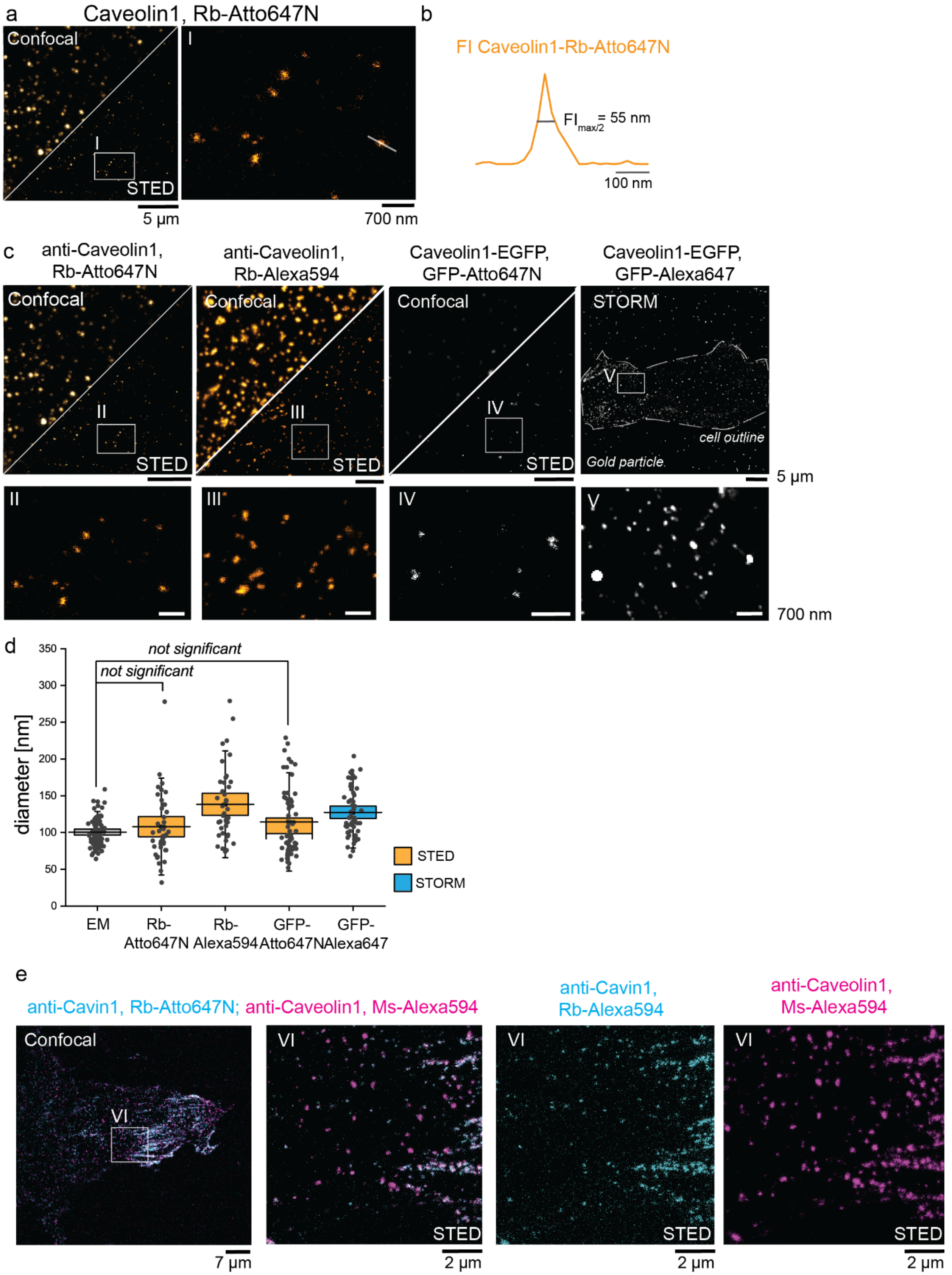
a
Caveolin1, Rb-Atto647N
Confocal
I
STED
5 µm
700 nm
b
FI Caveolin1-Rb-Atto647N
FI max/2 = 55 nm
100 nm
c
anti-Caveolin1, Rb-Atto647N
anti-Caveolin1, Rb-Alexa594
Caveolin1-EGFP, GFP-Atto647N
Caveolin1-EGFP, GFP-Alexa647
Confocal
STORM
II
III
IV
V
cell outline
Gold particle
STED
5 µm
700 nm
d
not significant
not significant
diameter [nm]
350
300
250
200
150
100
50
0
EM
Rb-Atto647N
Rb-Alexa594
GFP-Atto647N
GFP-Alexa647
STED
STORM
e
anti-Cavin1, Rb-Atto647N; anti-Caveolin1, Ms-Alexa594
anti-Cavin1, Rb-Alexa594
anti-Caveolin1, Ms-Alexa594
Confocal
VI
STED
7 µm
2 µm

Suppl. Figure 4: Exact caveolae size measurement by STED microscopy.

(a) Representative STED image of endogenous caveolin1 immunolabelled and tagged by Atto647N (Rabbit-Atto647N) in MEF plasma membrane sheets. Enlarged selection (a) illustrates single caveolin1 spots. White line indicates plot profile shown in (b, 3 independent experiments).

(b) STED fluorescence plot profile (in orange) of a caveolin1 spot. $FI_{max/2}$ = half maximum of fluorescence intensity spot.

(c) Representative caveolin1 fluorescence images obtained by STED and STORM (Stochastic Optical Reconstruction Microscopy). Lower panel illustrates zoom selection of the individual STED or STORM images.

(d) Caveolin1 spot size measurement based on fluorescence plot profiles. STED (orange) and STORM (blue) data was compared to diameter measured in PREM images (Fig. 1B). Box plot indicates mean ± SE, whiskers illustrate SD, each replicate is depicted (n(EM) = 82 caveolae, n(STED-Rb-Atto647N) = 41 spots, n(STED-Rb-Alexa594) = 42 spots, n(STED-GFP nanobody-Atto647N) = 65 spots, n(STORM-Alexa647) = 59 spots, 2 independent experiments). Statistical difference was calculated by two-sided Mann Whitney test.

(e) Representative confocal and STED image of endogenous cavin1 immunolabelled and tagged by Atto647N (Rabbit-Atto647N) in MEF plasma membrane sheets (cyan), caveolin1 was stained with antibody tagged with Alexa594 (mouse-Alexa594, magenta). Enlarged section represents STED image (VI, 2 independent experiments).

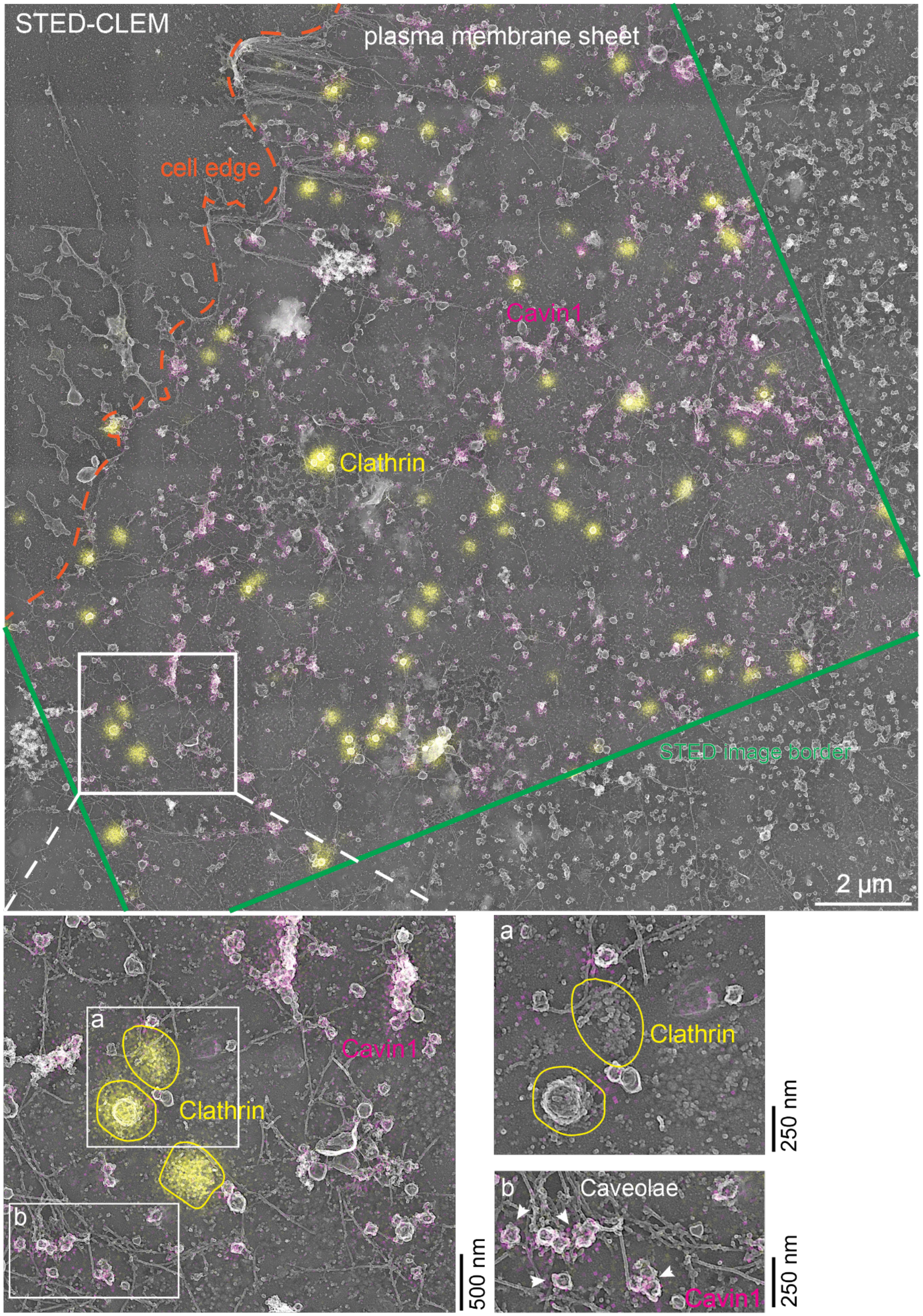
STED-CLEM
plasma membrane sheet
cell edge
Cavin1
Clathrin
STED image border
2 µm
a
b
Cavin1
Clathrin
500 nm
a
Clathrin
250 nm
b
Caveolae
Cavin1
250 nm

Suppl. Figure 5: STED-CLEM overview of plasma membrane sheets showing clathrin and cavin1.

Representative STED-CLEM image showing the acquired STED image that was correlated to the platinum replica TEM image. STED image border is shown in green, cell edge in orange. To achieve alignment of STED and PREM images, at first, cell edges were roughly aligned. Afterwards, detailed correlation was done by aligning clathrin clathrin fluorescence spots (in yellow) to the visible clathrin structures in the PREM image (done in Matlab, accordingly to[46,85]). Notably, due to the mass of the clathrin antibody, the characteristic geometric clathrin is partially obscured (see enlarged selection in a). Cavin1 fluorescence (in magenta) correlates to caveolae in PREM image (b, white arrows point to caveolae).

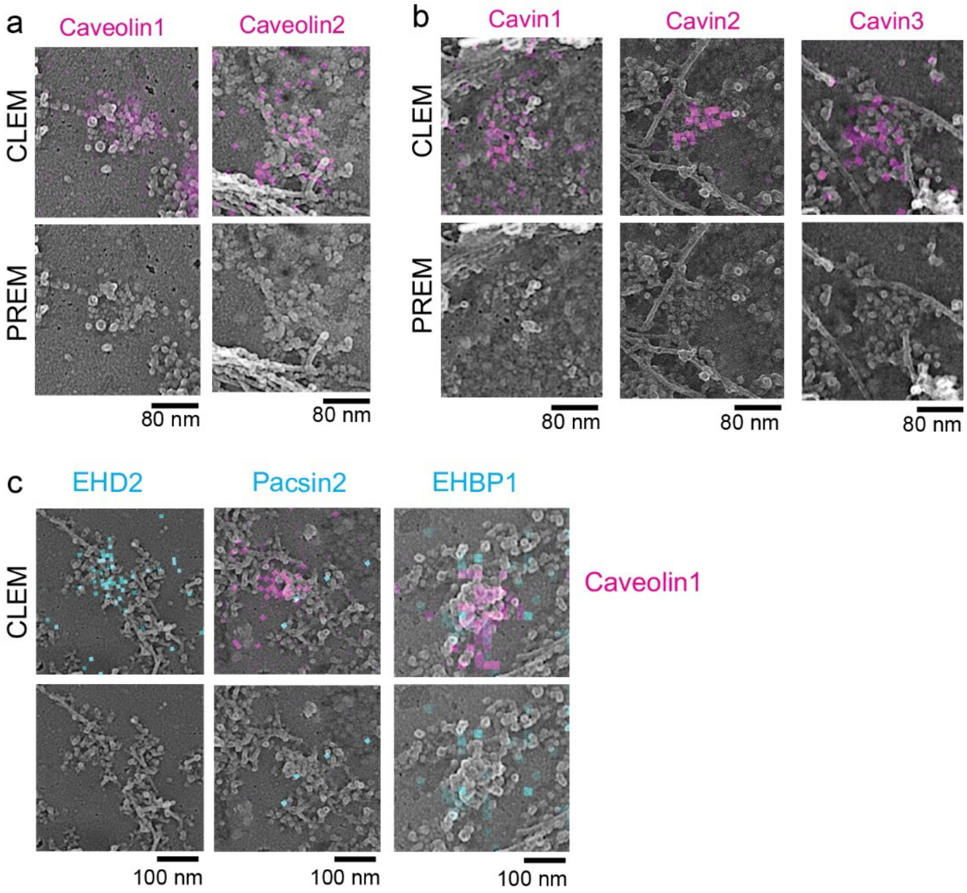

Suppl. Figure 6: Immature caveolae sites can be detected by STED-CLEM.

STED-CLEM of plasma membrane sheets of MEF expressing caveolin1 or 2 (a), cavin1-3 (b), or EHD2, pacsin2 or EHBP1 (c) revealed immature caveolae sites (3 independent experiments).

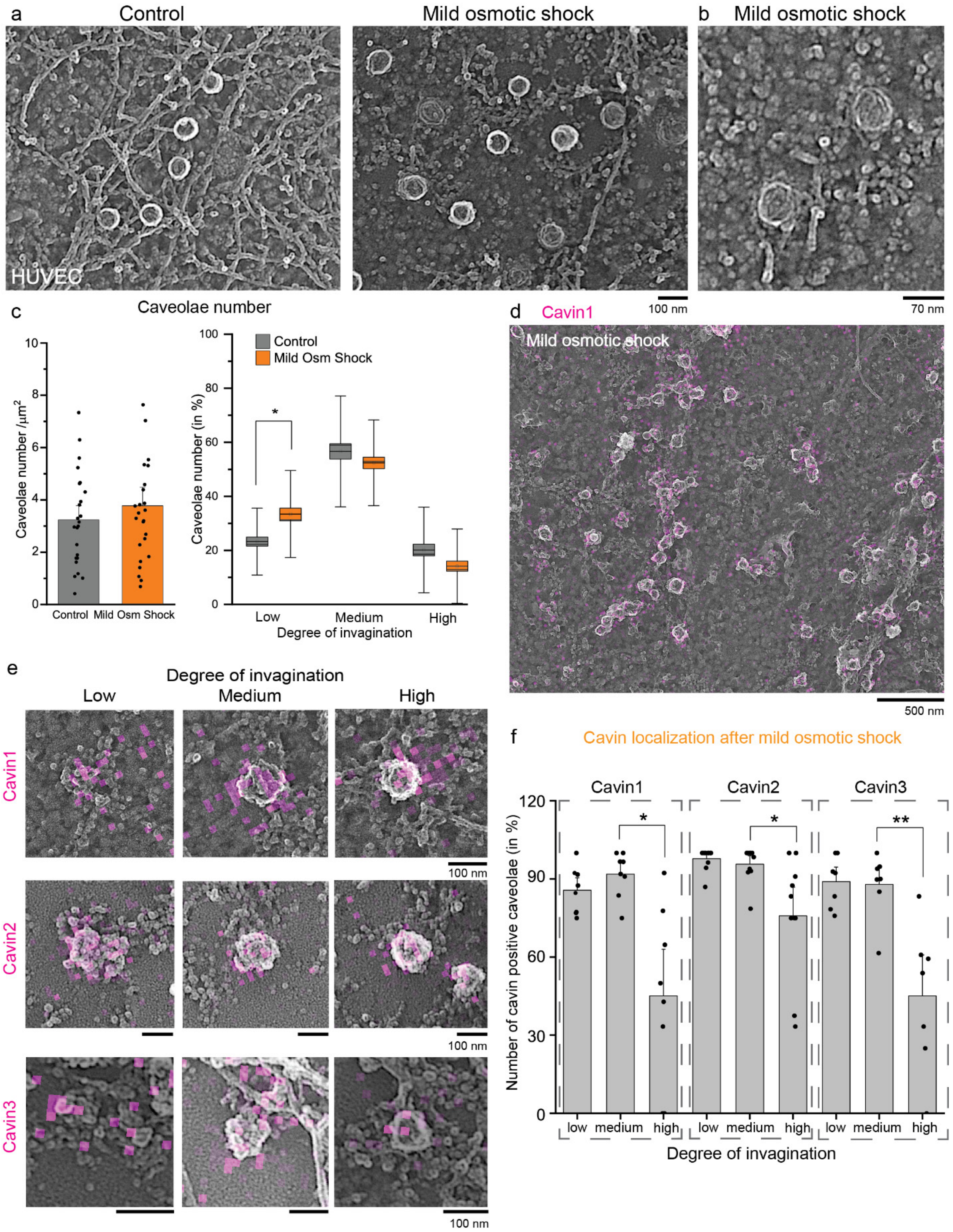
a
Control
Mild osmotic shock
b
Mild osmotic shock
HUVEC
100 nm
70 nm
c
Caveolae number
Caveolae number /µm²
Control
Mild Osm Shock
Caveolae number (in %)
Control
Mild Osm Shock
*
Low
Medium
High
Degree of invagination
d
Cavin1
Mild osmotic shock
500 nm
e
Degree of invagination
Low
Medium
High
Cavin1
Cavin2
Cavin3
100 nm
100 nm
100 nm
f
Cavin localization after mild osmotic shock
Cavin1
Cavin2
Cavin3
Number of cavin positive caveolae (in %)
*
*
**
low
medium
high
low
medium
high
low
medium
high
Degree of invagination

Suppl. Figure 7: Cavin localization at low curved caveolae after mild osmotic shock in HUVEC.

(a-b) Representative PREM images of caveolae in HUVEC plasma membrane sheets after mild osmotic shock (1:5 dilution with deionized water, 5 min).

(c) Caveolae number at the plasma membrane after mild osmotic shock (n(control) = 24 cell regions, n(mild osmotic) = 25 cell regions; Control: n(low) = 647 caveolae, n(medium) = 2053 caveolae, n(high) = 553 caveolae; Mild osmotic shock: n(low) = 581 caveolae, n(medium) = 1028 caveolae, n(high) = 246 caveolae, 3 independent experiments, * P = 0.011).

(d) Representative STED-CLEM image of endogenous cavin1 (magenta) after mild osmotic shock.

(e) STED-CLEM of cavin1, 2 or 3 (magenta) in different caveolae types after mild osmotic shock.

(f) Percentage of cavin positive caveolae in STED-CLEM images after mild osmotic shock (Cavin1: n(low) = 192 caveolae, n(medium) = 316 caveolae, n(high) = 65 caveolae/8 cell regions, * P = 0.0053; Cavin2: n(low) = 240 caveolae, n(medium) = 532 caveolae, n(high) = 89 caveolae/10 cell regions, * P = 0.0148; Cavin3: n(low) = 149 caveolae, n(medium) = 180 caveolae, n(high) = 92 caveolae/7 cell regions, ** P = 0.00329; 3 independent experiments).

Box or bar plots illustrate mean values ± SE, whiskers show SD, each replicate is depicted, two-sided Mann Whitney test for statistical difference.

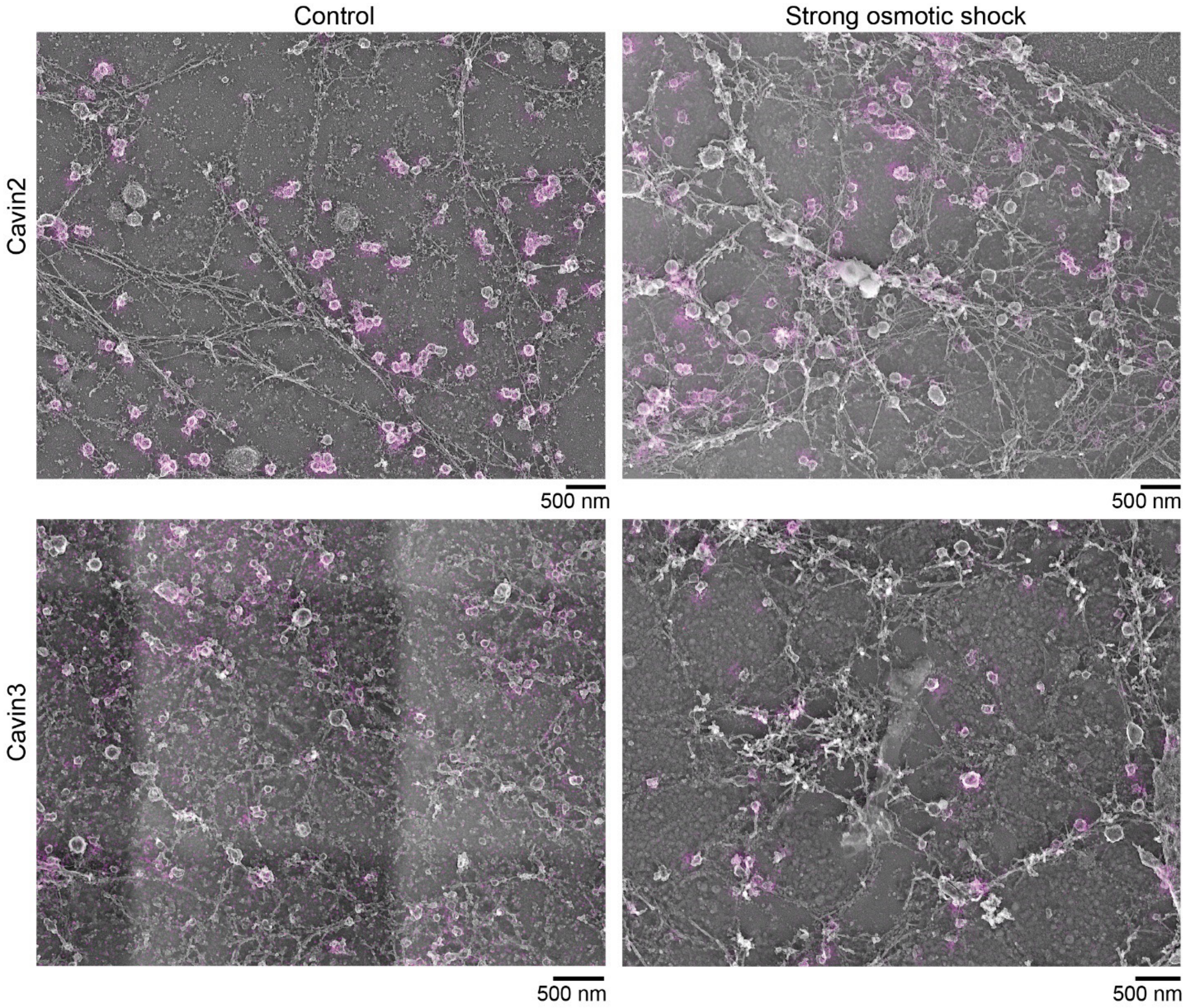

Suppl. Figure 8: Representative overview STED-CLEM images of endogenously stained cavin2 or 3 in HUVEC plasma membrane sheets treated with strong osmotic shock (1:9 dilution with deionized water, 3 independent experiments).

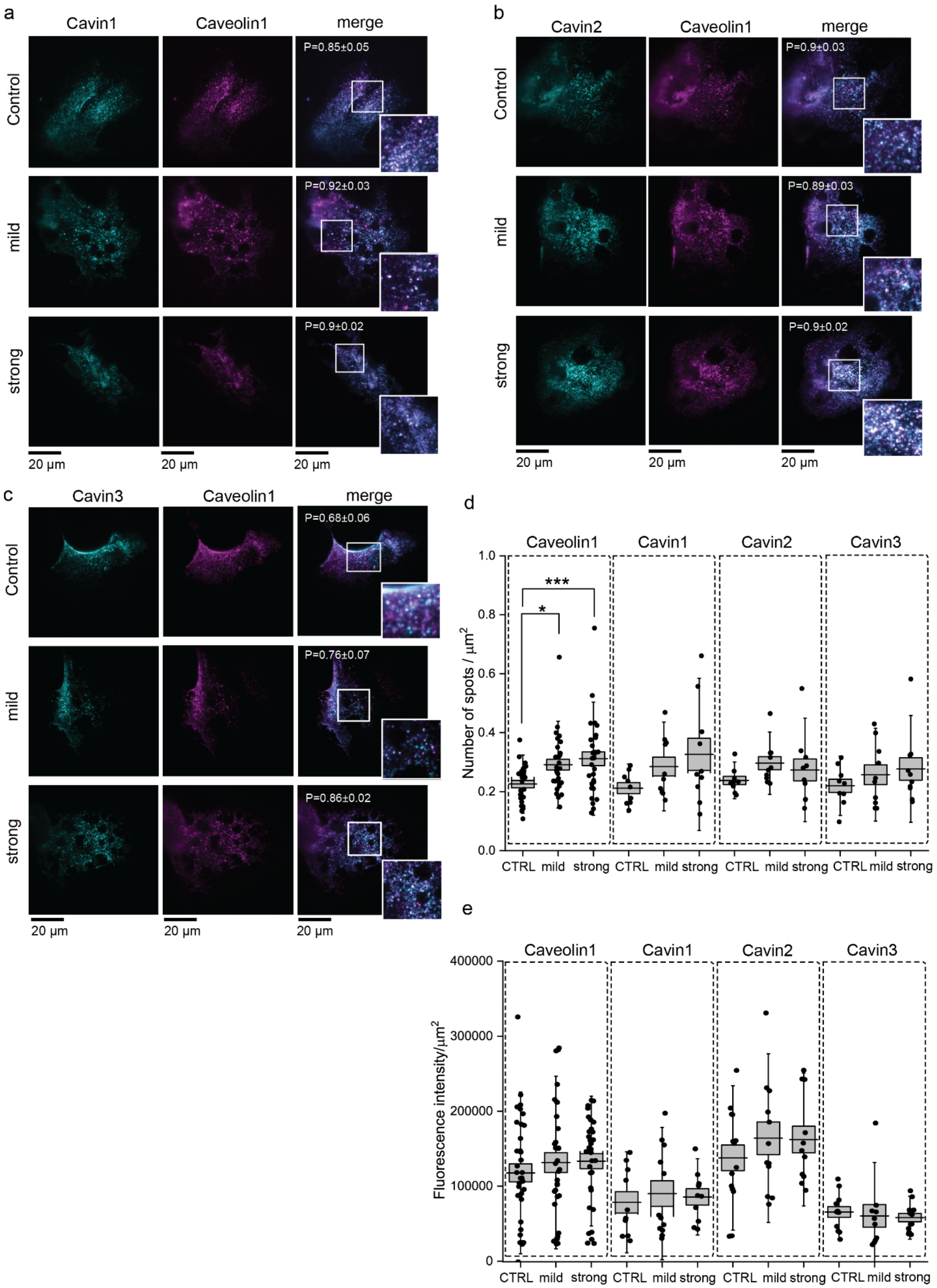

a
Cavin1
Caveolin1
merge
Control
mild
strong
P=0.85±0.05
P=0.92±0.03
P=0.9±0.02
20 µm
20 µm
20 µm
b
Cavin2
Caveolin1
merge
Control
mild
strong
P=0.9±0.03
P=0.89±0.03
P=0.9±0.02
20 µm
20 µm
20 µm
c
Cavin3
Caveolin1
merge
Control
mild
strong
P=0.68±0.06
P=0.76±0.07
P=0.86±0.02
20 µm
20 µm
20 µm
d
Caveolin1
Cavin1
Cavin2
Cavin3
Number of spots / µm²
***
*
1.0
0.8
0.6
0.4
0.2
0.0
CTRL mild strong CTRL mild strong CTRL mild strong CTRL mild strong
e
Caveolin1
Cavin1
Cavin2
Cavin3
Fluorescence intensity/µm²
400000
300000
200000
100000
0
CTRL mild strong CTRL mild strong CTRL mild strong CTRL mild strong

Suppl. Figure 9: Cavin1, 2 and 3 localization to Caveolin1 in intact HUVEC after osmotic shock.

(a-c) Representative TIRF images of intact HUVEC treated either with mild (1:5 dilution with deionized water) or strong (1:9 dilution with deionized water) osmotic shock for 5 min followed by immunostaining of cavin1 (a), cavin2 (b), or cavin3 (c). Cavin - caveolin1 colocalization was evaluated by Pearson correlation (P).

(d) Number of caveolin1 and cavin1-3 spots in TIRF imaging zone of the cell. (Caveolin1: n(CTRL) = 27 cells, n(mild) = 30 cells, n(strong) = 30 cells; cavin1: n(CTRL) = 9 cells, n(mild) = 10 cells, n(strong) = 10 cells; cavin2: n(CTRL) = 9 cells, n(mild) = 10 cells, n(strong) = 10 cells; cavin3: n(CTRL) = 9 cells, n(mild) = 10 cells, n(strong) = 10 cells; 2 independent experiments, * P = 0.00552, *** P = 6.9E-4).

(e) Fluorescence intensity per membrane area of endogenously caveolin1 or cavin1-3 staining after mild or strong osmotic shock (Caveolin1: n(CTRL) = 36 cells, n(mild) = 34 cells, n(strong) = 33 cells; cavin1: n(CTRL) = 10 cells, n(mild) = 12 cells, n(strong) = 11 cells; cavin2: n(CTRL) = 14 cells, n(mild) = 12 cells, n(strong) = 11 cells; cavin3: n(CTRL) = 12 cells, n(mild) = 10 cells, n(strong) = 12 cells; 2 independent experiments).

Box plots illustrate mean values ± SE, whiskers show SD, each replicate is depicted, two-sided Mann Whitney test for statistical difference.

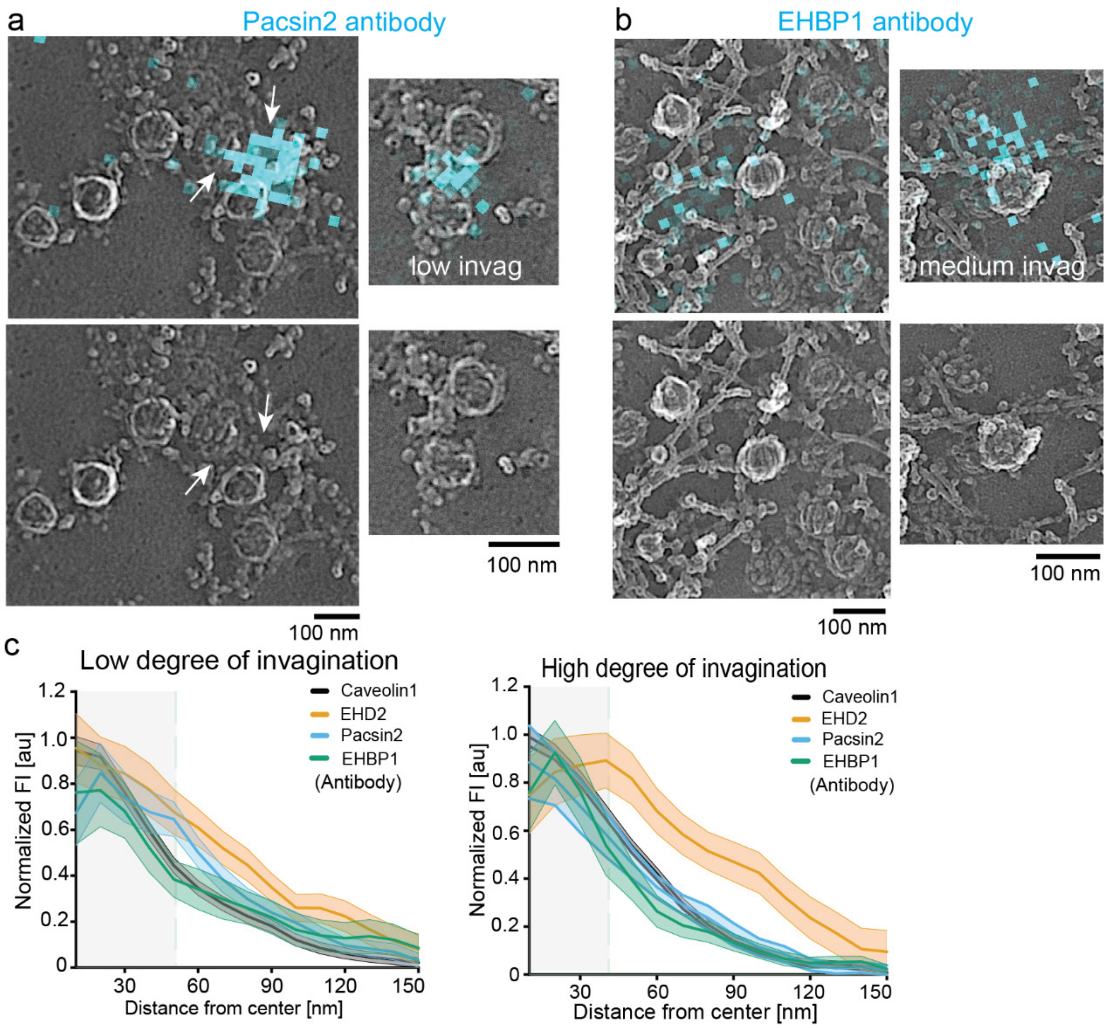

Suppl. Figure 10: Endogenous staining of Pacsin2 and EHBP1 at caveolae.

(a-b) Representative CLEM images for MEFs stained with antibodies against pacsin2 (A) or EHBP1 (B). The secondary antibody was labelled with Atto647N. White arrows in (A) indicate pacsin2 localization at low curved caveolae. Scale bar shows 80 nm.

(c) Quantitative analysis of low or highly curved caveolae by STED fluorescence profiles obtained from endogenous antibody staining. Line graphs show mean ± SE from the center of caveolae to their edges (indicated by green dashed line, caveolae number: caveolin1: n(low) = 145 , n(high) = 178 ; EHD2: n(low) = 165, n(high) = 161; pacsin2: n(low) = 77, n(high) = 49; EHBP1: n(low) = 60, n(high) = 37, 2 independent experiments).

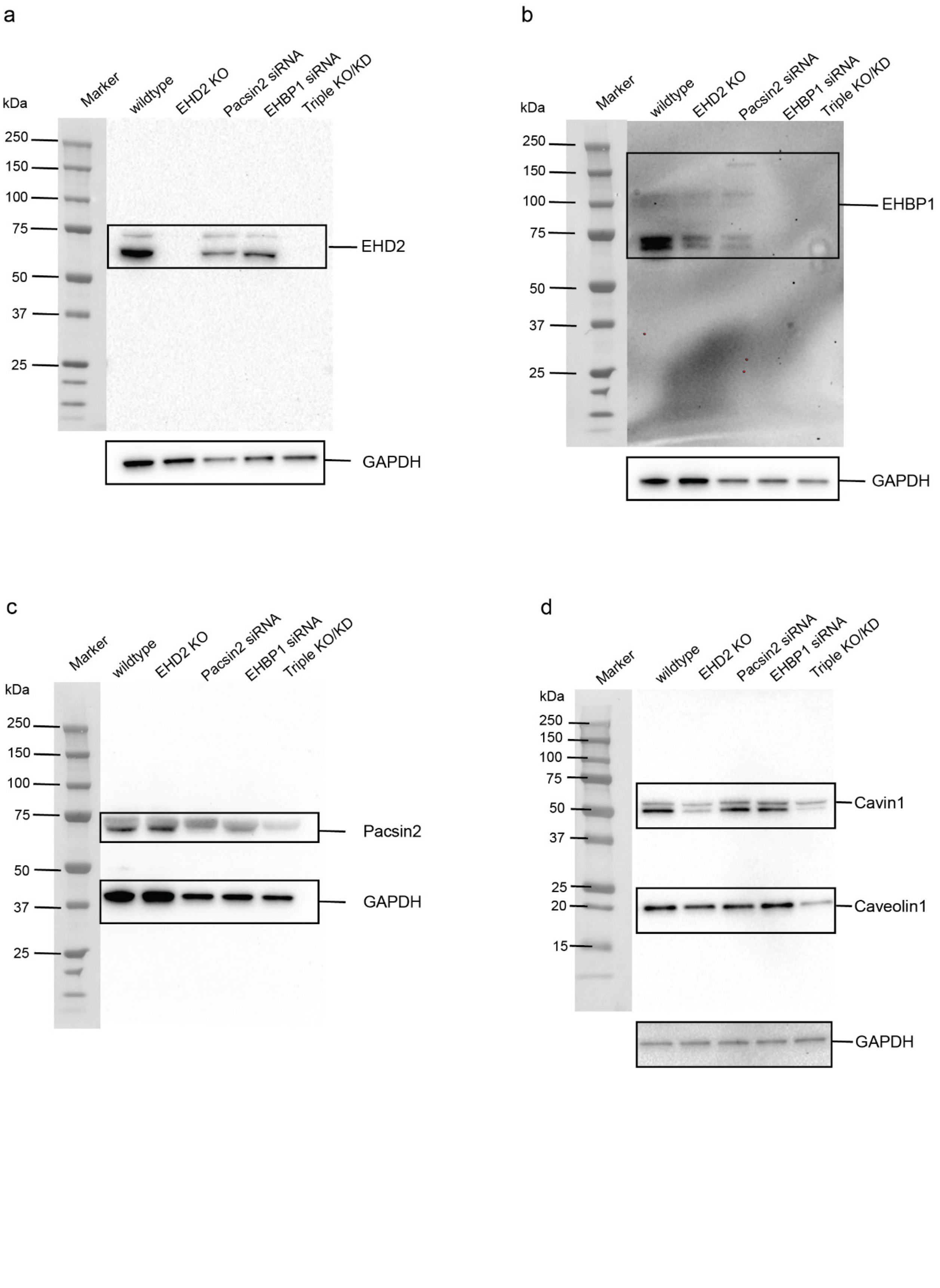
a
Marker
wildtype
EHD2 KO
Pacsin2 siRNA
EHBP1 siRNA
Triple KO/KD
kDa
250
150
100
75
50
37
25
EHD2
GAPDH
b
Marker
wildtype
EHD2 KO
Pacsin2 siRNA
EHBP1 siRNA
Triple KO/KD
kDa
250
150
100
75
50
37
25
EHBP1
GAPDH
c
Marker
wildtype
EHD2 KO
Pacsin2 siRNA
EHBP1 siRNA
Triple KO/KD
kDa
250
150
100
75
50
37
25
Pacsin2
GAPDH
d
Marker
wildtype
EHD2 KO
Pacsin2 siRNA
EHBP1 siRNA
Triple KO/KD
kDa
250
150
100
75
50
37
25
20
15
Cavin1
Caveolin1
GAPDH

Suppl. Figure 11: Western Blot validation of pacsin2 and EHBP1 knockdown.

(a-c) Western Blot validation of MEFs treated with pacsin2 or EHBP1 siRNA or EHD2 knockout (KO) MEFs showing protein levels of EHD2 (a), EHBP1 (b) or pacsin2 (c). EHBP1 antibody band size ~ 160-180 kDa, pacsin2 antibody band size ~ 60-65 kDa, GAPDH band size ~37 kDa (3 independent experiments).

(d) Western Blot analysis of cavin1 and caveolin1 protein levels in MEFs lacking EHD2, pacsin2 or EHBP1. Cavin1 antibody predicted band size ~ 51 kDa, caveolin1 band size ~ 20 kDa (3 independent experiments).

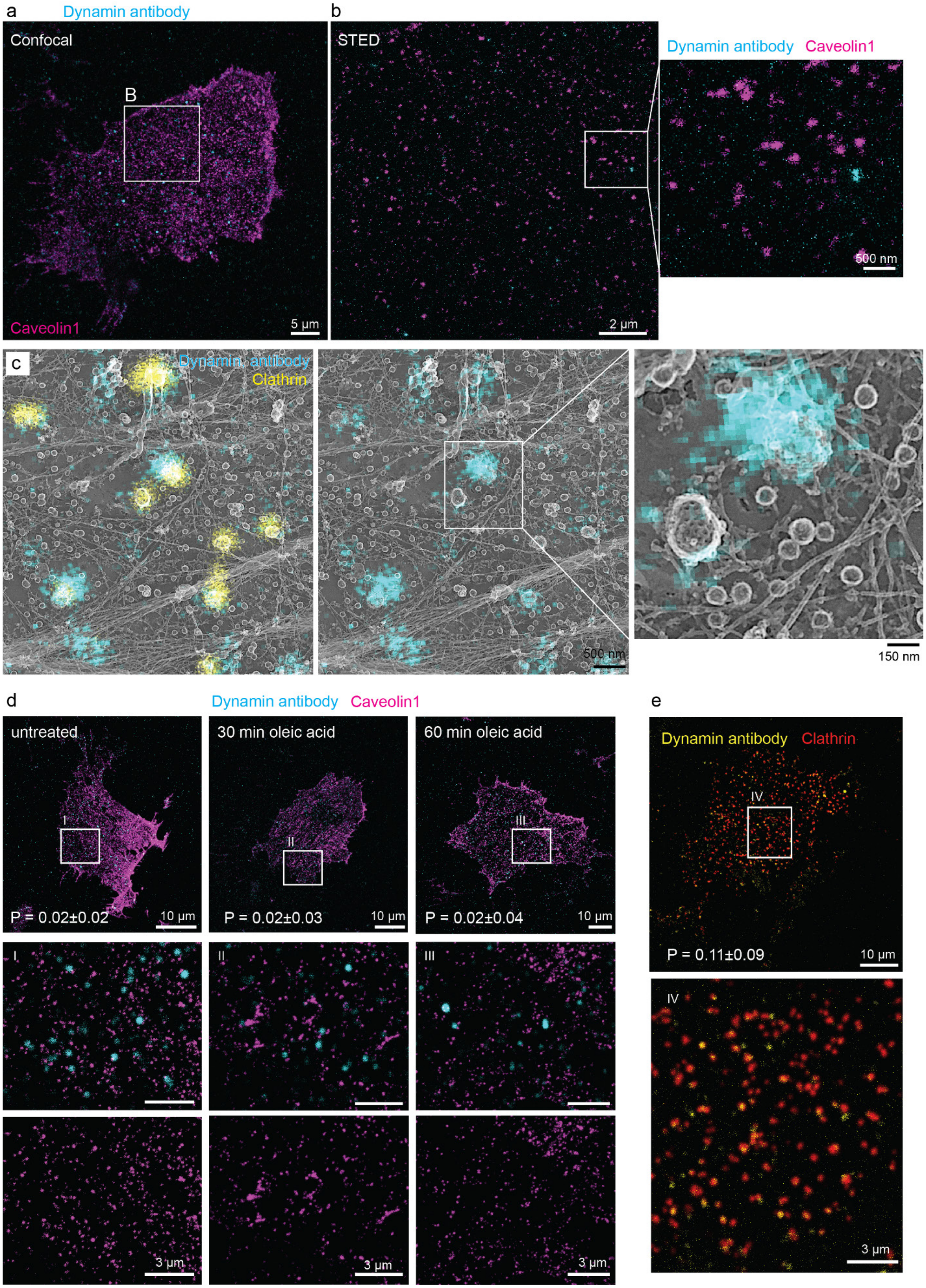
a
Dynamin antibody
Confocal
B
Caveolin1
5 µm
b
STED
2 µm
Dynamin antibody
Caveolin1
500 nm
c
Dynamin antibody
Clathrin
500 nm
150 nm
d
Dynamin antibody
Caveolin1
untreated
30 min oleic acid
60 min oleic acid
I
II
III
P = 0.02±0.02
P = 0.02±0.03
P = 0.02±0.04
10 µm
3 µm
e
Dynamin antibody
Clathrin
IV
P = 0.11±0.09
10 µm
3 µm

Suppl. Figure 12: Immunostaining of endogenous dynamin in MEFs did not show localization to caveolae.

(a) Confocal overview of MEF plasma membrane sheet stained against caveolin1 (magenta) and dynamin (cyan). Indicated selection was used for STED microscopy in B (2 independent experiments).
(b) Representative STED image of caveolin1 (magenta) and dynamin (cyan) labelled with antibodies.
(c) STED-PREM image showing antibody labelled dynamin (cyan) and clathrin (yellow, 2 independent experiments). Enlarged panel illustrates caveolae and specific dynamin localization to clathrin coat.
(d) Representative STED images of caveolin1 (magenta) and dynamin (cyan) labelled with antibodies in MEFs treated oleic acid for either 30 or 60 min. Pearson correlation (P) indicates caveolin1 – dynamin colocalization (n(untreated) = 9 cells, n(30 min) = 11 cells, n(60 min) = 9 cells, 2 independent experiments).
(e) Representative STED image of clathrin (red) and dynamin (yellow) labelled with antibodies illustrates colocalization (n =12 cells).

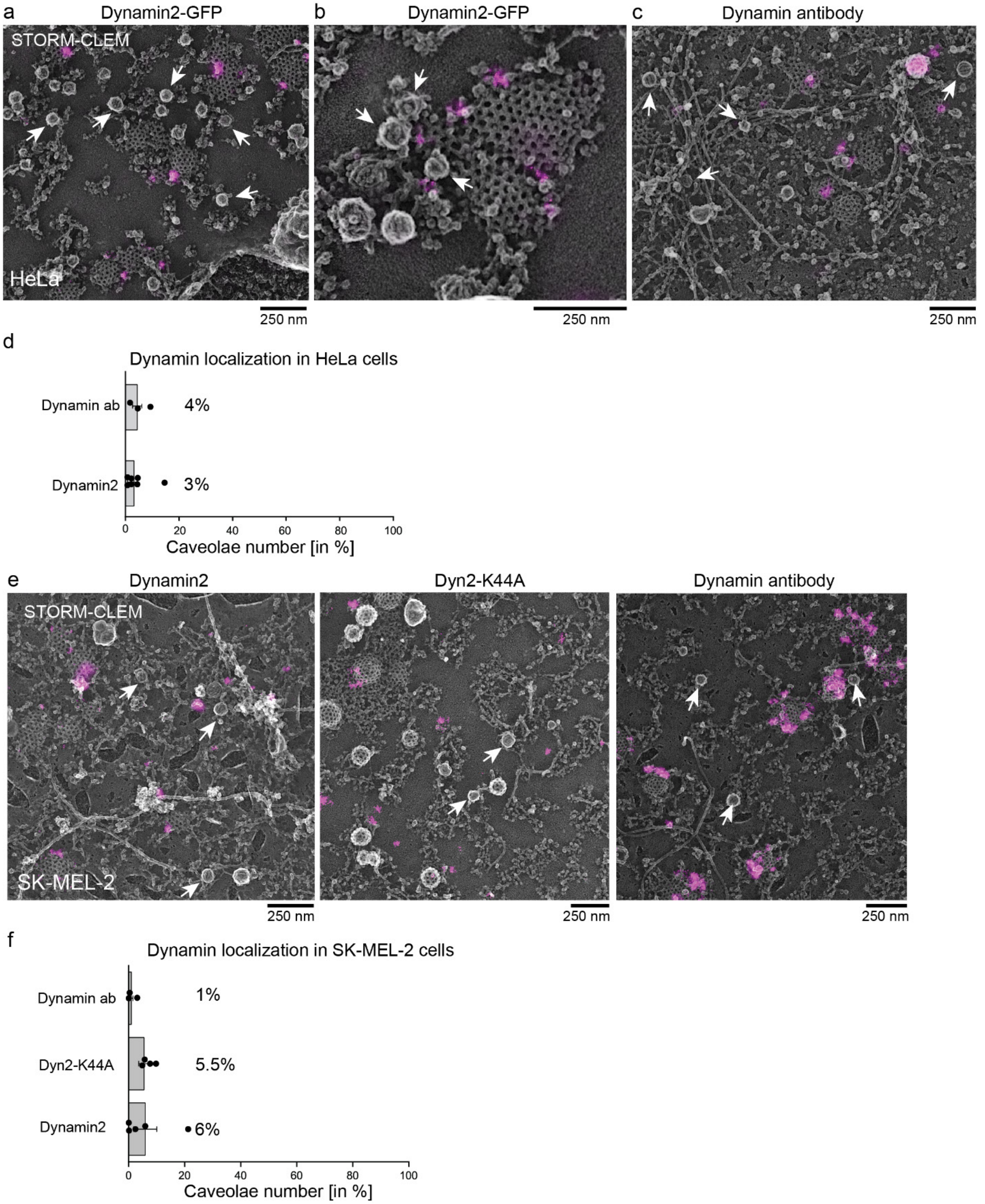
a
Dynamin2-GFP
STORM-CLEM
HeLa
250 nm
b
Dynamin2-GFP
250 nm
c
Dynamin antibody
250 nm
d
Dynamin localization in HeLa cells
Dynamin ab
4%
Dynamin2
3%
0
20
40
60
80
100
Caveolae number [in %]
e
Dynamin2
Dyn2-K44A
Dynamin antibody
STORM-CLEM
SK-MEL-2
250 nm
250 nm
250 nm
f
Dynamin localization in SK-MEL-2 cells
Dynamin ab
1%
Dyn2-K44A
5.5%
Dynamin2
6%
0
20
40
60
80
100
Caveolae number [in %]

Suppl. Figure 13: STORM-CLEM in HeLa and SK-MEL-2 cells did not show dynamin localization to caveolae.

(a-b) Representative STORM-CLEM images illustrating STORM signal (in magenta) of dynamin2-GFP in Pt replicas of HeLa plasma membrane sheets. (B) illustrates dynamin localization to clathrin coat in close proximity to caveolae. White arrows indicate caveolae.

(c) Representative STORM-CLEM images illustrating STORM signal (in magenta) of endogenous dynamin antibody labelling in Pt replicas of HeLa plasma membrane sheets. White arrows indicate caveolae.

(d) Percentage of caveolae that were targeted by either dynamin2 or dynamin antibody in HeLa. 75.3% of dynamin stained caveolae are close to clathrin (less than 50 nm distance). Bar graph shows mean ± SE (n(Dynamin antibody) = 428 caveolae/3 cells, n(Dynamin2) = 1649 caveolae/7 cells; 3 independent experiments).

(e) Representative STORM-CLEM images illustrating STORM signal (in magenta) in SK-MEL-2 cells expressing either dynamin2-GFP or Dyn2-K44A-GFP, or were immune-stained to label endogenous dynamin. White arrows indicate caveolae.

(f) Percentage of caveolae that were targeted by either dynamin2, dynamin2-K44A or dynamin antibody in SK-MEL-2 cells. Notably, 23.75% of dynK44A positive caveolae are close to clathrin (less than 50 nm distance). Bar graph shows mean ± SE (n(Dynamin antibody) = 428 caveolae/4 cells, n(Dyn2-K44A) = 270 caveolae/4 cells , n(Dynamin2) = 426 caveolae/5 cells; 3 independent experiments).

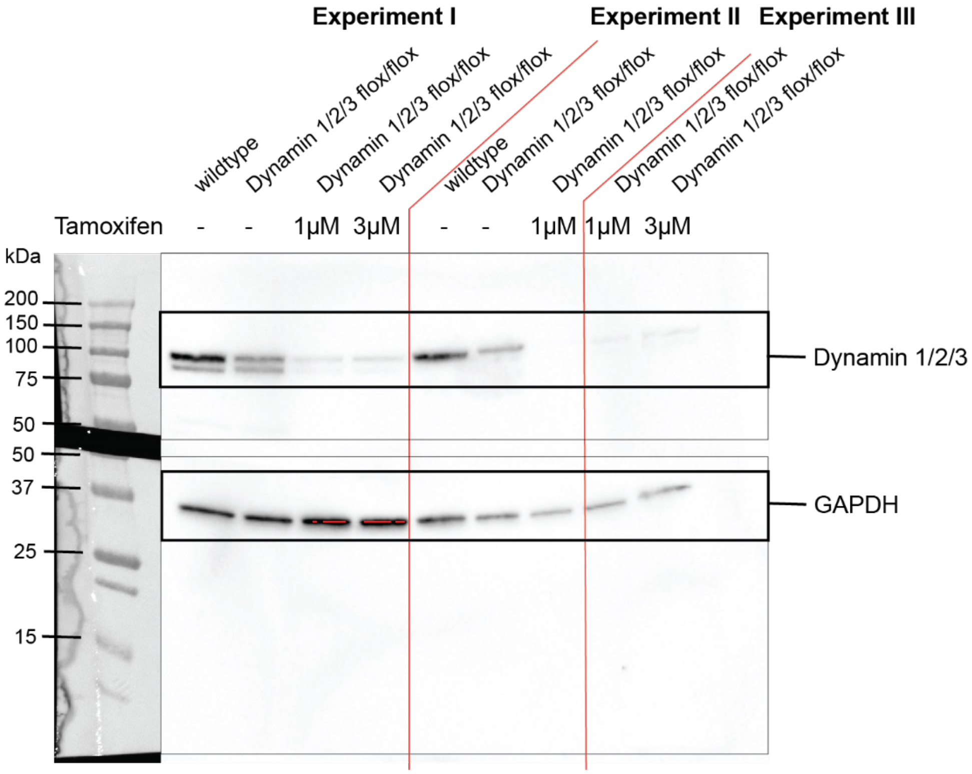

Suppl. Figure 14: Western Blot validation of dynamin triple knockout.

Western Blot illustrates three independent dynamin knockout experiments. Dynamin antibody recognizes all three isoforms, band size ~ 100 kDa, GAPDH band size 37 kDa (3 independent experiments).
