# Supplementary material for: The molecular organization of differentially curved caveolae indicates bendable structural units at the plasma membrane
Source: Nat Commun. 2022 Nov 24;13:7234. doi: 10.1038/s41467-022-34958-3 (PMC9700719; doi:10.1038/s41467-022-34958-3)
Supplement: Supplementary file 3 — Description of Additional Supplementary files [file 41467_2022_34958_MOESM3_ESM.pdf]

Description of Additional Supplementary Files

File name: Supplementary Movie 1
Description: Electron tomogram of MEF plasma membrane sheet including caveolae.

File name: Supplementary Movie 2
Description: Electron tomogram of low curved caveolae.

File name: Supplementary Movie 3
Description: Electron tomogram of medium curved caveolae.

File name: Supplementary Movie 4
Description: Electron tomogram of highly curved caveolae.
